# Supplementary material for: Relationship between ultra-widefield optical coherence tomography and ophthalmoscopy for detecting posterior inflammation in posterior uveitis and panuveitis
Source: PLoS One. 2023 Feb 10;18(2):e0281714. doi: 10.1371/journal.pone.0281714 (PMC9916645; doi:10.1371/journal.pone.0281714)
Supplement: Attachment — Submitted filename: Reviewer Attachments for Manuscript Number PONE-D-22-28716.docx [file pone.0281714.s002.docx]

1. line 73: 2 comma
2. line 197: Uveitis is known to increase IOP due to clogging of the trabecular meshwork with inflammatory cells, trabeculitis, pupillary block, synechial closure or secondary to steroid usage. However, decrease aqueous humor production may cause lower IOP initially. Lower IOP was observed in “W-OCT cells” group, yet was not observed in "Ophthalmoscopic findings " detected cells group. Try to clarify the reasons or mechanism in discussion.
3. line 206: They reported that anterior-chamber inflammation… Please list the author’s name.
